# Population, economic and geographic predictors of nations' medal tallies at the Pyeongchang and Tokyo Olympics and Paralympics

**DOI:** 10.3389/fspor.2022.931817

**Published:** 2022-08-17

**Authors:** Feifei Li, Will G. Hopkins, Patrycja Lipinska

**Affiliations:** ^1^Centre for Health and Exercise Science Research, Department of Sport, Physical Education, and Health, Hong Kong Baptist University, Hong Kong, China; ^2^Institute for Health and Sport, Victoria University, Melbourne, VIC, Australia; ^3^Institute of Physical Education, Kazimierz Wielki University, Bydgoszcz, Poland

**Keywords:** athletes, competitions, medals, Muslim, Olympics, Paralympics, performance, prediction

## Abstract

**Purpose:**

Ranking of nations by medal tally is a popular feature of the Olympics, but such ranking is a poor measure of sporting prowess or engagement until the tallies are adjusted for major factors beyond the control of individual nations. Here we estimate and adjust for effects of total population, economy expressed as gross domestic product per capita, absolute latitude and Muslim population proportion on total medal counts in female, male, mixed and all events at the Pyeongchang winter and Tokyo summer Olympics and Paralympics.

**Methods:**

The statistical model was multiple linear over-dispersed Poisson regression. Population and economy were log-transformed; their linear effects were expressed in percent per percent units and evaluated in magnitude as the factor effects of two between-nation standard deviations (SD). The linear effect of absolute latitude was expressed and evaluated as the factor effect of 30° (approximately 2 SD). The linear effect of Muslim proportion was expressed as the factor effect of 100% vs. 0% Muslim. Nations were ranked on the basis of actual vs. predicted all-events medal counts.

**Results:**

At the Pyeongchang Olympics, effects of population and economy were 0.7–0.8 %/% and 1.1–1.7 %/% (welldefined extremely large increases for 2 SD), factor effects of 30° of latitude were 11–17 (welldefined extremely large increases), and factor effects of 100% Muslim population were 0.08–0.69 (extremely large to moderate reductions, albeit indecisive). Effects at the Tokyo Olympics were similar in magnitude, including those of latitude, which were surprisingly still positive although diminished (large to very large increases). Effects at the Pyeongchang and Tokyo Paralympics were generally similar to those at the Olympics, but the effects of economy were diminished (large to very large increases). After adjustment of medal tallies for these effects, nations that reached the top-10 medalists in both winter games were Austria, Belarus, Kazakhstan, Slovakia and Ukraine, but only Azerbaijan reached the top-10 in both summer games.

**Conclusion:**

Adjusting medal counts for demographic and geographic factors provides a comparison of nations' sporting prowess or engagement that is more in keeping with the Olympic ideal of fair play and more useful for nations' Olympic-funding decisions.

## Introduction

Ranking of nations based on medal tallies is an interesting feature of the Olympics, but such a ranking is a poor measure of sporting prowess or engagement until the tallies are adjusted for major factors beyond the control of individual nations. Much of the popular media analysis of Olympic medal tallies is superficial, and academic efforts to move beyond this are vital to understanding structures, advantages and impediments to sports throughout the world. Appropriate adjustment is also important for nations whose Olympic-funding decisions include consideration of performance of their athletes relative to that of other nations.

Of the numerous studies of factors affecting nations' medal tallies in the summer Olympics, the most comprehensive is that of Grancay and Dudas (2018). After noting that population and gross domestic product (GDP) per capita were consistent predictors in previous research, these authors assembled a set of 144 potential predictors and used correlations among them to select population, GDP per capita, host country (prior, current and future), a civil-liberties index and absolute latitude as the main predictors of various measures of medal success in each Olympics between 2000 and 2016. They summarized the magnitudes of the effects by stating that “coefficients in the main regression equations have the anticipated signs and with a minor exception are all statistically significant.” The exception was civil liberties, with a non-significant negative effect (more liberties, less medals). The participation of women in Summer and Winter Olympics has been increasing since their first participation in 1900 (Wikipedia, 2022) until Tokyo 2020, where women accounted for a record 48% of the athletes [International Olympic Committee (IOC), 2022a]. Multiple new mixed-gender events have also been added to further promote gender equality, with Tokyo 2020 hosting 18 mixed events [International Olympic Committee (IOC), 2022b]. Given the rise in female participation in Olympic sports, Bredtmann et al. (2016) included an indicator variable for “country that has predominantly Muslim population” in a prediction model for medal tallies at the 2016 Olympics using data for the Olympics between 1996 and 2012, as these countries tended to send fewer athletes and won fewer medals in women's events. Nevertheless, magnitudes of the “Muslim proportion” effect and of the other effects in their model were not presented or assessed. People who follow the ways and life style of Islam are called Muslim, and participation in sports is not against the spirit of Islam and sharia law, which is the Islamic code of life (Marwat et al., 2014). Therefore, the reality is complex, with multiple reasons potentially contributing to the marginalization of Muslim women athletes; for example, maintenance of the hijaab (covering of the hair and other parts of the body) may be a barrier to participation in sports (Benn and Dagkas, 2013). The most recent study of nations' medal tallies is that of Belli and Saracoglu (2021), who correlated medal counts with population and GDP of the 20 nations with the highest medal counts at Tokyo. We could find only one study of factors affecting medal tallies in the winter Olympics: Johnson and Ali (2004) found GDP per capita was more important than population in the winter Olympics, but the relative importance reversed in the summer Olympics.

The Paralympic Games have received much less attention in respect of factors affecting medal tallies. Indeed, only two publications on Paralympics medal tallies are comparable to those on Olympics. Vanlandewijck et al. (2007) found that nation surface area, GDP per capita, and population were significant predictors of medal points at the Athens 2004 summer Paralympic and Olympic Games, with greater variance explained at the Olympic. Buts et al. (2013) performed a more comprehensive analysis of the summer Paralympics from Atlanta 1996 to Beijing 2008; they reported statistically significant effects of GDP per capita, population, participants per population, nation surface area, nation mean temperature, former Communist-bloc nation, games host, and former games host.

With the increase in the participation of women and in the number of mixed-gender team events at the Pyeongchang winter and Tokyo summer Olympics and Paralympics, we considered that a re-evaluation of factors affecting nations' medal winning at these games was timely, with separate analyses for male, female, mixed and all events. We have focused on a model that includes the main predictors in previous studies: population, GDP per capita and latitude. Mean temperature would be another obvious predictor, but Grancay and Dudas (2018) found that latitude was effectively a proxy for mean temperature, so we did not include it. We have also followed the lead of Bredtmann et al. (2016) by including proportion of Muslims in each nation's population as a predictor. We predicted medal counts rather than the weighted medal points (gold ×3 + silver ×2 + bronze ×1) of some previous authors, since in our view the differences in performance between gold, silver and bronze are not sufficient to merit such weighting. Modeling medal counts rather than proportion of total medal counts or total medal points, as some previous authors have recommended, also allows a more practical assessment of magnitudes of effects in terms of proportional (factor or ratio) increases or decreases in the counts. For our focus on factors beyond the control of individual nations, we opted not to include the number of participants sent to the Games, which would in any case be confounded by the ability of the athletes. We also omitted a Communist-bloc predictor, which would be a crude measure for many nations and would have a waning influence on recent games. Surface area would give an unreasonable disadvantage to some nations (e.g., Canada, Australia and Brazil) and an unreasonable advantage to others (e.g., Hong Kong and other small island nations) after adjustment, so it was also omitted. The effects of hosting the games could not be estimated from a single winter and summer games, but we expected Korea and Japan to perform well after adjustment for the predictors we included. Finally, we have ranked nations by assessing their actual medal counts relative to their predicted medal counts after adjustment.

## Materials and methods

The lists of nations participating at the Pyeongchang and Tokyo games were obtained from Wikipedia (2018, 2021a). Medal tallies were obtained from a database maintained by the Institute for Applied Training Science (2018, 2021) of the University of Leipzig. Population and GDP per capita were obtained from the relevant pages of the website of the World Bank (2021) for years beginning the Olympic quadrennium (2015 for the Pyeongchang, 2017 for the Tokyo). Mean latitudes were obtained from the website World Map (2021). Proportions of Muslims were obtained from Wikipedia (2021b). Variables of income inequality (GINI index) reported by The World Bank and gender gap score assessed by World Economic Forum Global Gender Gap Report were extracted and analyzed. But due to the limited number of countries available and missing data, both were omitted from the analyses.

Data were analyzed with the Statistical Analysis System (SAS On Demand for Academics, version 9.4, SAS Institute, Cary NC). Medal counts were predicted with an over-dispersed Poisson regression model (Berdahl et al., 2015; Grancay and Dudas, 2018) realized with the generalized linear mixed model procedure (Proc Glimmix) using a log link. Separate analyses were performed for male, female, mixed and all events with the Pyeongchang and with the Tokyo data. Over-dispersion factors ranged from 2.3 for all events down to 0.41 (under-dispersion) for mixed events at the Pyeongchang Olympics, and from 3.9 for all events down to 1.4 for mixed events at the Tokyo Olympics. These factors represent and account for a tendency of medals to occur in clusters within nations for all but the mixed events at Pyeongchang. Over-dispersion factors showed a similar pattern but larger values across the event groups at the Pyeongchang Paralympics (4.2 in all events down to 0.4 in mixed events) and at the Tokyo Paralympics (8.1 down to 1.0).

The predictors were log of population, log of GDP per capita, absolute latitude, and proportion of Muslims in the population. The coefficients of population and GDP per capita in the model were interpreted directly as percent differences in medal count per percent difference in the predictor; they were also multiplied by two between-nation standard deviations (SD) of the log-transformed variables and back-transformed to factor effects per SD^2^ (per 2 SD of the log-transformed variable) for assessment of their magnitudes (Hopkins et al., 2009). The coefficient for absolute latitude was back-transformed and expressed as the factor effect per 30°, which is ~2 SD of this variable. The coefficient for Muslim proportion was back-transformed and expressed as the factor effect of 100% Muslim relative to 0% Muslim. Plots of standardized residuals vs. each predictor showed no evidence of heteroscedasticity suggestive of non-linearity in any analysis. Residuals in the analyses, representing differences between observed and predicted medal counts for medaling nations, were back-transformed to factors for ranking the nations (best = largest factor >1; worst = smallest factor <1); nations winning no medals were ranked on the basis of predicted medal counts (worst = highest predicted count).

The qualitative magnitude of the back-transformed effects and residuals was assessed with the generic scale for factor effects on counts: threshold ratios of 0.9. 0.7. 0.5, 0.3, and 0.1 for small, moderate large, very large and extremely large reductions, and their inverses 1.11, 1.43, 2.0, 3.3, and 10 respectively for increases (Hopkins, 2010); these thresholds correspond to 1 in 10 through 9 in 10 medals being due to application of a negative or positive effect. Sampling uncertainty for effects is presented as 90% compatibility limits, in ± form for effects in percent per percent units and in ×/÷ form for factor effects. Sampling uncertainty was evaluated using substantial and non-substantial hypothesis tests and using reference-Bayesian probabilities of substantial and trivial effects for a minimally informative prior (Hopkins, 2020). Thresholds for assessing magnitudes of correlations between nations' ranks at the Olympics and Paralympics were 0.1, 0.3, 0.5, 0.7 and 0.9 for small through extremely large (Hopkins, 2010). Sampling uncertainty in the correlations could not be derived simply from sample size, owing to interdependency of the individual ranks arising from the prediction equations, so the correlations are treated as case-study values at a winter and summer games.

## Results

### Pyeongchang and Tokyo Olympics

Descriptive statistics for the nations' medal counts, population, GDP per capita, latitude and Muslim proportion are shown in Table 1 for the Pyeongchang Olympics and in Table 2 for the Tokyo Olympics. Back-transformed means and factor SD derived from log-transformed values are shown for population and GDP, since log-transformed values were used in the Poisson regression, and the magnitude of these effects is expressed per two SD of the log-transformed values (per the factor SD^2^).

**Table 1:** Descriptive statistics of the 91 nations that attended the Pyeongchang winter Olympics in 2018.

	**Mean ±SD or Mean ×/÷SD**	**Range**
Medals
All events	3.4 ± 7.6	0 to 39
Male events	1.7 ± 4.1	0 to 24
Female events	1.5 ± 3.2	0 to 12
Mixed events	0.2 ± 0.8	0 to 5
Population (×10^6^)
Raw	61 ± 200	0.03 to 1,370
Back-transformed^a^	9.4 ×/÷ 8.5	0.03 to 1,370
GDP (US$, ×10^3^)
Raw	25 ± 31	0.42 to 167
Back-transformed^a^	12 ×/÷ 3.8	0.42 to 167
Latitude (°)		
Raw	32 ± 25	−41 to 65
Absolute	37 ± 16	0 to 65
Muslims (%)	17 ± 29	0 to 99

a The standard deviation (SD) is a times/divide factor.

Population and gross domestic product per capita (GDP) statistics are for the year 2015.

**Table 2:** Descriptive statistics of the 205 nations that attended the Tokyo summer Olympics in 2021.

	**Mean ±SD or Mean ×/÷SD**	**Range**
Medals
All events	5.3 ± 14.1	0 to 113
Male events	2.6 ± 6.3	0 to 41
Female events	2.4 ± 7.2	0 to 66
Mixed events	0.3 ± 1.1	0 to 8
Population (×10^6^)
Raw	37 ± 141	0.01 to 1,420
Back-transformed^a^	4.9 ×/÷ 10	0.01 to 1,420
GDP (US$, ×10^3^)
Raw	16 ± 25	0.29 to 171
Back-transformed^a^	6.4 ×/÷ 4.3	0.29 to 171
Latitude (°)		
Raw	19 ± 24	−41 to 65
Absolute	25 ± 17	0 to 65
Muslims (%)	25 ± 36	0 to 100

a The standard deviation (SD) is a times/divide factor.

Population and gross domestic product per capita (GDP) statistics are for the year 2017.

Table 3 shows the effects of population, GDP per capita, absolute latitude and proportion of Muslims on medal counts in all, male, female and mixed events provided by Poisson regression for the Pyeongchang and Tokyo Olympics. Population had similar and somewhat <1:1 proportional effects on medal winning (slopes of 0.60–0.84 %/%) at both Olympics, and these translated into similar extremely large decisive difference in medal counts between nations differing by 2 SD (factor SD^2^) of population, with the greatest differences for the mixed events. GDP showed more than a 1:1 proportional effects for the winter Olympics (although decisively more than 1:1 only for the mixed events), and again these translated into extremely large decisive difference at both Olympics, especially for the mixed events. Comparison of the compatibility limits shows that the estimates for the summer Olympics were more precise than those for the winter Olympics.

**Table 3:** Effects of population, gross domestic product per capita (GDP), absolute latitude and proportion of Muslims on medal counts in all, male, female and mixed events provided by multiple linear Poisson regression for the Pyeongchang winter Olympics and the Tokyo summer Olympics.

	**All**	**Male**	**Female**	**Mixed**
**Pyeongchang winter Olympics**
Population
Percent per percent	0.66, ±0.10	0.66, ±0.13	0.64, ±0.12	0.83, ±0.20
Factor per SD^2^	17, ×/÷1.6 E.large↑^****^	17, ×/÷1.8 E.large↑^****^	15, ×/÷1.7 E.large↑^****^	35, ×/÷2.3 E.large↑^****^
GDP
Percent per percent	1.12, ±0.24	1.08, ±0.30	1.09, ±0.28	1.72, ±0.52
Factor per SD^2^	20, ×/÷1.9 E.large↑^****^	18, ×/÷2.3 E.large↑^****^	18, ×/÷2.1 E.large↑^****^	99, ×/÷3.9 E.large↑^****^
Absolute latitude
Factor per 30°	11, ×/÷1.7 E.large↑^****^	11, ×/÷2.0 E.large↑^****^	11, ×/÷1.9 E.large↑^****^	17, ×/÷2.6 E.large↑^****^
Muslim proportion
Factor 100%/0%	0.10, ×/÷15 V.large↓	0.10, ×/÷30 V.large↓	0.08, ×/÷26 E.large↓	0.70, ×/÷50 Moderate↓
**Tokyo summer Olympics**
Population
Percent per percent	0.66, ±0.06	0.60, ±0.07	0.69, ±0.07	0.84, ±0.18
Factor per SD^2^	21, ×/÷1.3 E.large↑^****^	17, ×/÷1.4 E.large↑^****^	25, ×/÷1.4 E.large↑^****^	51, ×/÷2.3 E.large↑^****^
GDP
Percent per percent	0.55, ±0.12	0.46, ±0.13	0.62, ±0.14	0.88, ±0.36
Factor per SD^2^	4.9, ×/÷1.4 V.large↑^****^	3.8, ×/÷1.5 V.large↑^****^	5.9, ×/÷1.5 V.large↑^****^	13, ×/÷2.8 E.large↑^****^
Absolute latitude
Factor per 30°	2.5, ×/÷1.3 Large↑^****^	2.7, ×/÷1.3 Large↑^****^	2.2, ×/÷1.4 Large↑^****^	4.1, ×/÷2.2 V.large↑^****^
Muslim proportion
Factor 100%/0%	0.43, ×/÷1.7 Large↓^***^	0.58, ×/÷1.7 Moderate↓^**^	0.29, ×/÷2.1 V.large↓^***^	0.11, ×/÷22 V.large↓

The effects of population and GDP are shown as their regression coefficients (slopes, in %/% units) and, for evaluation of magnitude, as the slopes multiplied by two between-nation standard deviations (SD) of the log-transformed predictor (expressed as a factor per SD^2^, after back-transformation).

E, extremely; V, very; GDP, gross domestic product; ↑, increase; ↓, decrease.

Magnitude thresholds for small, moderate, large, very large and extremely large factors: 1.11, 1.43, 2.0, 3.3, 10 for increases; 0.9, 0.7, 0.5, 0.3, 0.1 for decreases.

Reference-Bayesian likelihoods of substantial effects: *possibly; **likely; ***very likely; ****most likely; *** and **** indicate rejection of the non-superiority or non-inferiority hypothesis (p_N−_ or p_N+_ < 0.05 and < 0.005 respectively).

All effects shown with likelihoods had adequate precision at the 99% level (i.e., rejection of the inferiority or superiority hypothesis, p_−_ or p_+_ < 0.005), with the exception of Muslim proportion on male medals at Tokyo (adequate at the 90% level, i.e., rejection of the superiority hypothesis, p_+_ < 0.05).

Medal counts for countries 30° closer to the Poles were much higher: extremely large differences at the winter Olympics, and somewhat smaller but still large to very large differences at the summer Olympics, again with the largest effects for the mixed events. Nations with 100% Muslim population had much lower medal counts than those with 0% Muslims, but the effects were indecisive at the winter Olympics and indecisive for the mixed events at the summer Olympics. The biggest observed reduction in medal counts in Muslim nations was for the female winter and summer events and the mixed summer events.

**Figure 1:**
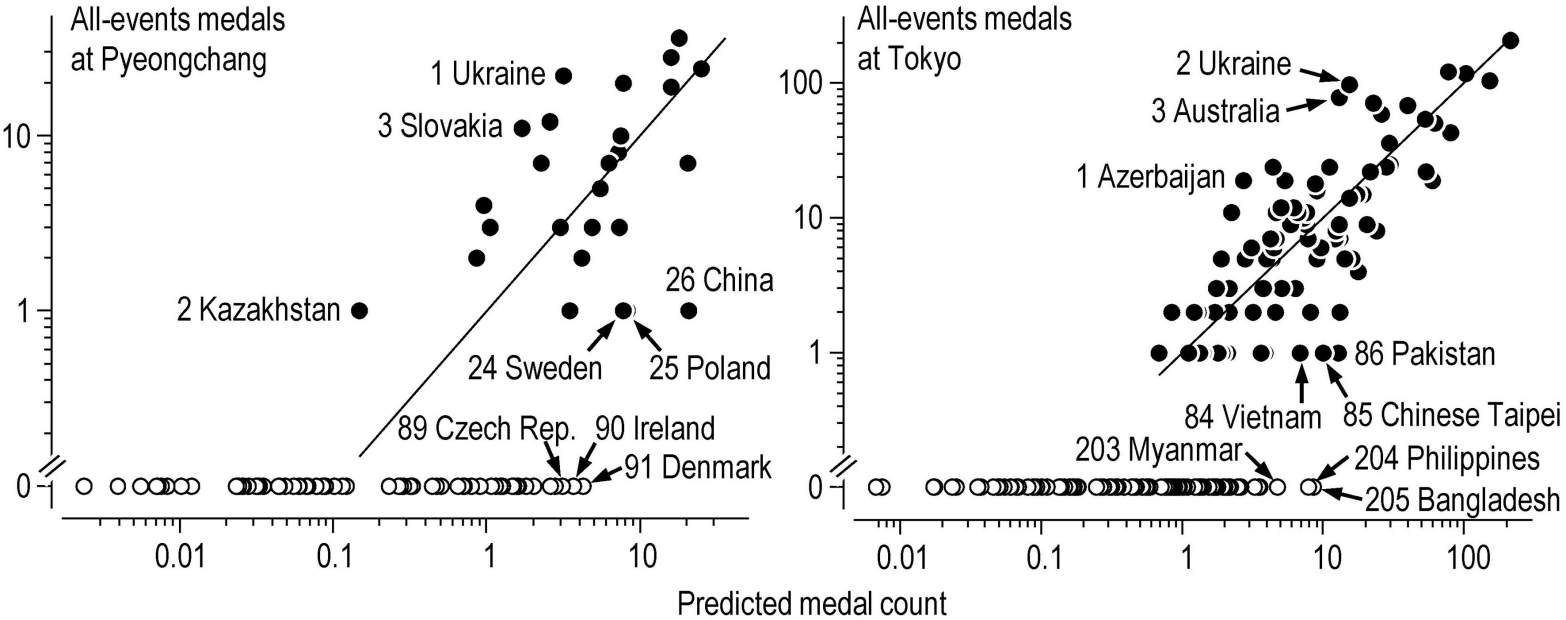
All-events medal counts and medal counts predicted by the Poisson regression model at the Pyeongchang and Tokyo Olympics. Filled and open symbols are values for individual nations that won respectively at least one medal and no medals. For nations that won medals, the numbers on the labels represent the ranking based on the medal count as a factor of the predicted medal count (the vertical distance between the actual and predicted medal count, represented by the line of identity, in these log-log graphs). Ranking of nations that won no medals is based on the predicted medal count. Labels indicate the best three medalists, the worst three medalists, and the worst three non-medalists.

Tables 4, 5 show top-10 and bottom-10 nation rankings based on differences between actual medal counts and medal counts predicted by population, GDP, absolute latitude and Muslim proportion. The full lists of rankings, including those for male, female and mixed events, are available in a supplementary Excel workbook **Olympics nation ranks.xlsx**. The rankings are illustrated graphically in log-log plots (Figure 1). Noteworthy rankings at the winter Olympics are the top position of host-nation Korea with 4× more medals than predicted (a very large increase above the predicted value), China at rank 21 (with slightly more than the predicted number of medals), the United States at rank 25 (with small=moderate fewer medals than the predicted number), Great Britain at the bottom of the medalists (a very large reduction compared with predicted), and Denmark and Ireland with no medals instead of the predicted 8 or 9. At the summer Olympics, the host nation Japan did not make the top-10 (it was rank 46), Jamaica and San Marino were top of a group of three nations with very large increases above predicted medals, Finland was at the bottom of the medal table with a borderline extremely large reduction below predicted medals, and Chile had the highest predicted medal count of the non-medalists.

**Table 4:** Ranking of the best medalists, worst medalists and worst non-medalists at the Pyeongchang winter Olympics.

**Rank**	**Nation**	**Population (×10^6^)**	**GDP** **(US$ ×10^3^)**	**Latitude (°)**	**Muslims (%)**	**All medals**	**Predicted medals**	**Ratio all/** **predicted**
**Best medalists**
1	Korea	76	20	38	0.1	17	4.2	4.0
2	Austria	8.6	44	48	8.0	14	4.4	3.2
3	Belarus	9.5	5.9	54	0.8	3	1.0	3.1
4	Kazakhstan	18	11	48	70	1	0.4	2.8
5	Slovenia	2.1	21	46	3.6	2	0.7	2.7
6	Czech Republic	11	18	50	0.2	7	2.6	2.7
7	Norway	5.2	74	60	3.2	39	18	2.2
8	Slovakia	5.4	16	49	0.2	3	1.4	2.1
9	Netherlands	17	45	52	5.1	20	11	1.8
10	Ukraine	45	2.1	48	1.7	1	0.6	1.8
**Worst medalists**
21	China	1,370	8.1	36	1.7	9	8.5	1.1
22	Sweden	9.8	52	60	8.1	14	15	0.9
23	Latvia	2.0	14	57	0.2	1	1.2	0.9
24	Hungary	9.8	13	47	0.5	1	1.4	0.7
25	United States	320	57	37	1.1	23	32	0.7
26	Finland	5.5	43	62	1.8	6	11	0.5
27	Spain	46	26	40	2.6	2	4.7	0.4
28	Poland	38	13	52	0.02	2	4.9	0.4
29	Belgium	11	41	51	7.6	1	6.2	0.2
30	Great Britain	65	45	55	6.3	5	33	0.2
**Worst non-medalists**
82	Greece	11	18	39	3.9	0	1.1	0
83	Portugal	11	19	39	0.4	0	1.2	0
84	Estonia	1.3	17	59	0.0	0	1.3	0
85	Lithuania	2.9	14	55	0.1	0	1.3	0
86	Romania	20	9.0	46	0.7	0	1.3	0
87	Argentina	43	14	−38	0.9	0	2.0	0
88	Luxembourg	0.6	101	50	2.3	0	2.5	0
89	Iceland	0.3	53	65	0.2	0	3.0	0
90	Ireland	4.7	62	53	1.4	0	8.0	0
91	Denmark	5.7	53	56	5.4	0	8.8	0

For medalists the ranking is highest to lowest values of the ratio all-events medals divided by medals predicted by population, gross domestic product per capita (GDP), absolute latitude and Muslim proportion; for non-medalists, the ranking is lowest to highest values of predicted medals.

**Table 5:** Ranking of the best medalists, worst medalists and worst non-medalists at the Tokyo summer Olympics.

**Rank**	**Nation**	**Population (×10^6^)**	**GDP** ** (US$ ×10^3^)**	**Latitude (°)**	**Muslims (%)**	**All medals**	**Predicted medals**	**Ratio all/** ** predicted**
**Best medalists**
1	Jamaica	2.9	5.1	18	0.2	9	0.7	12
2	San Marino	0.03	45	44	0.0	3	0.3	11
3	Grenada	0.1	10	12	0.3	1	0.1	9.5
4	Kenya	50	1.6	0	11	10	1.3	7.7
5	Fiji	0.9	6.1	−17	6.3	2	0.3	6.0
6	Cuba	11	8.5	22	0.1	15	2.6	5.8
7	Azerbaijan	9.8	4.1	40	97	7	1.2	5.6
8	Kyrgyzstan	6.2	1.2	41	85	3	0.5	5.6
9	Georgia	3.7	4.4	42	11	8	1.5	5.4
10	Uganda	41	0.7	1	14	4	0.8	5.2
**Worst medalists**
84	Romania	20	11	46	0.7	4	8.8	0.5
85	South Africa	57	6.1	−31	1.9	3	8.0	0.4
86	Thailand	69	6.6	16	5.4	2	5.9	0.3
87	Ireland	4.8	70	53	1.4	4	12	0.3
88	India	1,340	2.0	21	15	7	23	0.3
89	Mexico	125	9.3	24	0.0	4	14	0.3
90	Saudi Arabia	33	21	24	98	1	4.0	0.2
91	Lithuania	2.8	17	55	0.1	1	4.2	0.2
92	Argentina	44	15	−38	0.9	3	14	0.2
93	Finland	5.5	46	62	1.8	2	14	0.1
**Worst non-medalists**
196	Uruguay	3.4	19	−33	0.0	0	2.5	0
197	Iraq	38	5.1	33	96	0	2.7	0
198	Bangladesh	160	1.6	24	90	0	2.9	0
199	U.A. Emirates	9.5	41	23	76	0	3.1	0
200	Peru	31	6.7	−9	0.0	0	3.1	0
201	Iceland	0.3	72	65	0.2	0	3.1	0
202	Luxembourg	0.6	107	50	2.3	0	3.5	0
203	Pakistan	208	1.5	30	97	0	3.8	0
204	Vietnam	95	2.4	14	0.1	0	4.1	0
205	Chile	19	15	−36	0.0	0	7.4	0

For medalists the ranking is highest to lowest values of the ratio all-events medals divided by medals predicted by population, gross domestic product per capita (GDP), absolute latitude and Muslim proportion; for non-medalists, the ranking is lowest to highest values of predicted medals.

### Pyeongchang and Tokyo Paralympics

Descriptive statistics for the nations' medal counts, population, GDP per capita, latitude and Muslim proportion are shown in Table 6 for the Pyeongchang winter Paralympics and in Table 7 for the Tokyo summer Paralympics. Back-transformed means and factor SD derived from log-transformed values are shown for population and GDP, since log-transformed values were used in the Poisson regression, and the magnitude of these effects is expressed per two SD of the log-transformed values (per the factor SD^2^).

**Table 6:** Descriptive statistics of the 91 nations that attended the Pyeongchang winter Paralympics in 2018.

	**Mean ±SD or mean ×/÷SD**	**Range**
Medals
All events	2.6 ± 6.6	0 to 36
Male events	1.3 ± 3.5	0 to 22
Female events	1.2 ± 3.5	0 to 20
Mixed events	0.1 ± 0.5	0 to 4
Population (×10^6^)
Raw	61 ± 200	0.03 to 1,370
Back-transformed^a^	9.4 ×/÷ 8.5	0.03 to 1,370
GDP (US$, ×10^3^)
Raw	25 ± 31	0.42 to 167
Back-transformed^a^	12 ×/÷ 3.8	0.42 to 167
Latitude (°)		
Raw	32 ± 25	−41 to 65
Absolute	37 ± 16	0 to 65
Muslims (%)	17 ± 29	0 to 99

a The standard deviation (SD) is a times/divide factor.

Population and gross domestic product per capita (GDP) statistics are for the year 2015.

**Table 7:** Descriptive statistics of the 205 nations that attended the Tokyo summer Paralympics in 2021.

	**Mean ±SD or mean ×/÷SD**	**Range**
Medals
All events	8.1 ± 23.8	0 to 208
Male events	4.1 ± 11.1	0 to 83
Female events	3.4 ± 11.6	0 to 117
Mixed events	0.6 ± 1.8	0 to 15
Population (×10^6^)
Raw	37 ± 141	0.01 to 1,420
Back-transformed^a^	4.9 ×/÷ 10	0.01 to 1,420
GDP (US$, ×10^3^)
Raw	16 ± 25	0.29 to 171
Back-transformed^a^	6.4 ×/÷ 4.3	0.29 to 171
Latitude (°)		
Raw	19 ± 24	−41 to 65
Absolute	25 ± 17	0 to 65
Muslims (%)	25 ± 36	0 to 100

a The standard deviation (SD) is a times/divide factor.

Population and gross domestic product per capita (GDP) statistics are for the year 2017.

Table 8 shows the effects of population, GDP per capita, absolute latitude and proportion of Muslims on medal counts in all, male, female and mixed events provided by Poisson regression for the Pyeongchang and Tokyo Paralympics. Population had similar and somewhat less than 1:1 proportional effects on medal winning (slopes of 0.60–0.84 %/%) at both Paralympics, and these translated into similar extremely large decisive difference in medal counts between nations differing by 2 SD (factor SD^2^) of population, with the greatest differences for female events. GDP had generally lower proportional effects than did population (slopes of 0.37–0.69 %/%), and these translated into large to very large decisive differences at both Paralympics, with the greatest effects for the mixed events.

**Table 8:** Effects of population, gross domestic product per capita (GDP), absolute latitude and proportion of Muslims on medal counts in all, male, female, and mixed events provided by multiple linear Poisson regression for the Pyeongchang winter Paralympics and the Tokyo summer Paralympics.

	**All**	**Male**	**Female**	**Mixed**
**Pyeongchang winter Paralympics**
Population
Percent per percent	0.71, ±0.15	0.60, ±0.18	0.84, ±0.18	0.82, ±0.24
Factor per SD^2^	21, ×/÷1.9 E.large↑^****^	13, ×/÷2.2 E.large↑^****^	36, ×/÷2.1 E.large↑^****^	33, ×/÷2.8 E.large↑^****^
Gdp
Percent per percent	0.39, ±0.28	0.43, ±0.34	0.37, ±0.30	0.69, ±0.42
Factor per SD^2^	2.9, ×/÷2.1 Large↑^***^	3.2, ×/÷2.5 Large↑^***^	2.7, ×/÷2.2 Large↑^***^	6.4, ×/÷3.1 V.large↑^***^
Absolute latitude
Factor per 30°	13, ×/÷2.2 E.large↑^****^	6.7, ×/÷2.5 V.large↑^****^	27, ×/÷2.3 E.large↑^****^	21, ×/÷3.6 E.large↑^****^
Muslim proportion
Factor 100%/0%	0.01, ×/÷110 E.large↓	0.00, ×/÷820 E.large↓	0.03, ×/÷75 E.large↓	0.01, ×/÷4,400 E.large↓
**Tokyo summer paralympics**
Population
Percent per percent	0.76, ±0.07	0.73, ±0.08	0.82, ±0.08	0.66, ±0.09
Factor per SD^2^	35, ×/÷1.4 E.large↑^****^	30, ×/÷1.4 E.large↑^****^	46, ×/÷1.4 E.large↑^****^	21, ×/÷1.5 E.large↑^****^
Gdp
Percent per percent	0.39, ±0.13	0.34, ±0.14	0.43, ±0.14	0.53, ±0.18
Factor per SD^2^	3.1, ×/÷1.5 Large↑^****^	2.7, ×/÷1.5 Large↑^****^	3.4, ×/÷1.5 V.large↑^****^	4.6, ×/÷1.7 V.large↑^****^
Absolute latitude
Factor per 30°	3.1, ×/÷1.3 Large↑^****^	3.1, ×/÷1.4 Large↑^****^	3.2, ×/÷1.4 Large↑^****^	3.5, ×/÷1.5 V.large↑^****^
Muslim proportion
Factor 100%/0%	0.63, ×/÷1.7 Moderate↓^**^	0.79, ×/÷1.7 Small↓	0.50, ×/÷1.9 Moderate↓^**^	0.15, ×/÷3.1 V.large↓^****^

The effects of population and GDP are shown as their regression coefficients (slopes, in %/% units) and, for evaluation of magnitude, as the slopes multiplied by two between-nation standard deviations (SD) of the log-transformed predictor (expressed as a factor per SD^2^, after back-transformation).

E, extremely; V, very; ↑, increase; ↓, decrease.

Magnitude thresholds for small, moderate, large, very large and extremely large factors: 1.11, 1.43, 2.0, 3.3, 10 for increases; 0.9, 0.7, 0.5, 0.3, 0.1 for decreases.

Reference-Bayesian likelihoods of substantial effects: *possibly; **likely; ***very likely; ****most likely; *** and **** indicate rejection of the non-superiority or non-inferiority hypothesis (p_N−_ or p_N+_ < 0.05 and < 0.005 respectively).

All effects shown with likelihoods had adequate precision at the 99% level (i.e., rejection of the inferiority or superiority hypothesis, p_−_ or p_+_ < 0.005), with the exception of GDP on all, male and female medals at Pyeongchang and Muslim proportion on all and female medals at Tokyo (adequate at the 90% level, i.e., rejection of the inferiority or superiority hypothesis, p_−_ or p_+_ < 0.05).

Medal counts for nations 30° closer to the Poles were much higher: very large to extremely large differences at the winter Paralympics, and somewhat smaller but still large to very large differences at the summer Paralympics, with the largest effects for the female and mixed events. Nations with 100% Muslim population had much lower medal counts than those with 0% Muslims at the winter Paralympics, but the effects were indecisive; the reductions in medal counts due to 100% Muslim population were less marked at the summer Paralympics (small to very large) and indecisive only for the male events.

**Figure 2:**
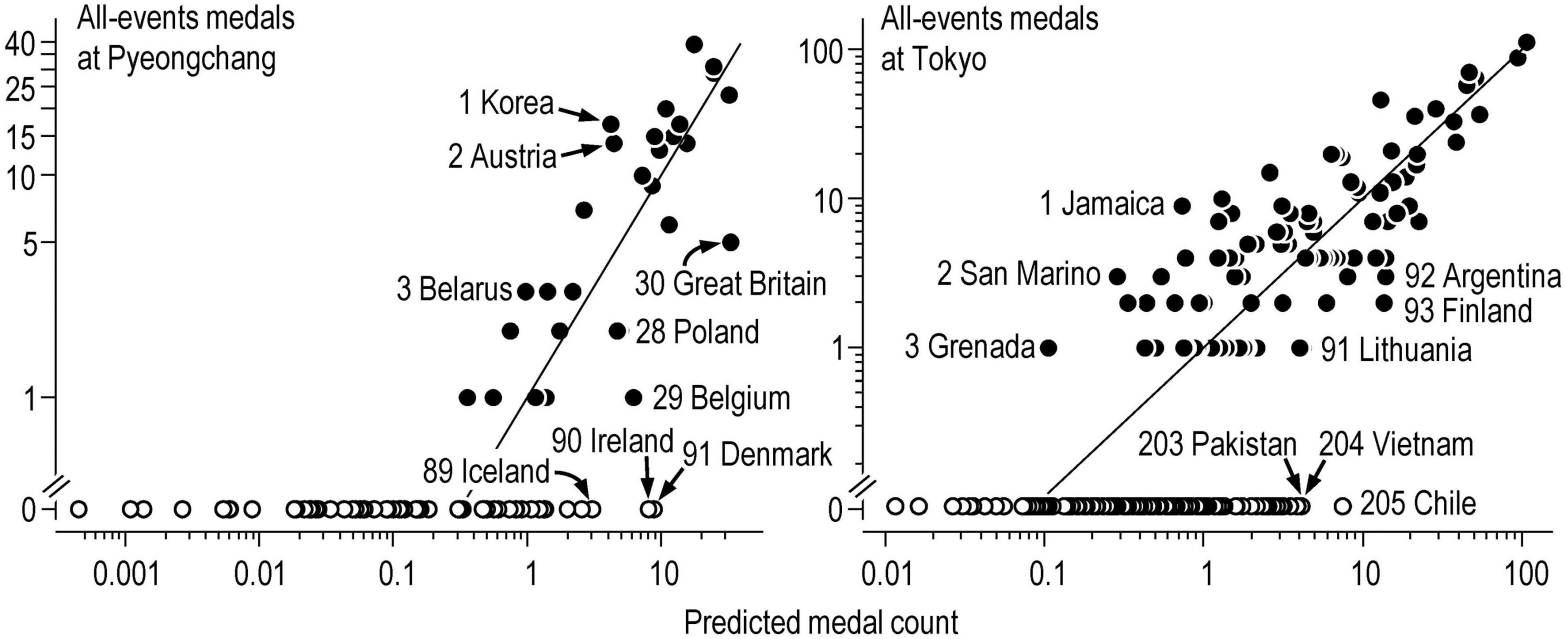
All-events medal counts and medal counts predicted by the Poisson regression model at the Pyeongchang and Tokyo Paralympics. Filled and open symbols are values for individual nations that won respectively at least one medal and no medals. For nations that won medals, the numbers on the labels represent the ranking based on the medal count as a factor of the predicted medal count (the vertical distance between the actual and predicted medal count, represented by the line of identity, in these log-log graphs). Ranking of nations that won no medals is based on the predicted medal count. Labels indicate the best three medalists, the worst three medalists, and the worst three non-medalists.

Tables 9, 10 show top-10 and bottom-10 nation rankings based on differences between actual medal counts and medal counts predicted by population, GDP, absolute latitude and Muslim proportion. The full lists of rankings, including those for male, female and mixed events, are available in a supplementary Excel workbook **Paralympics nation ranks.xlsx**. The all-events rankings are illustrated graphically in log-log plots (Figure 2). Noteworthy rankings at the winter Paralympics are the top three medalist nations Ukraine, Kazakhstan and Slovakia, with more than 6x their predicted medal counts, the absence of host nation Korea from the top 10, the United States at rank 10 with twice their predicted medal count, China at the bottom of the medalists with only 5% of their predicted count, and Ireland and Denmark with no medals instead of their predicted ~4. At the summer Paralympics, the host nation Japan did not make the top-10, six of the top-10 nations had high proportions of Muslims (89–99%), Chinese Taipei and Pakistan were at the bottom of the medal table with ~10% of their predicted medals, while the Philippines and Bangladesh had no medals instead of their predicted ~8.

**Table 9:** Ranking of the best medalists, worst medalists and worst non-medalists at the Pyeongchang winter Paralympics.

**Rank**	**Nation**	**Population (×10^6^)**	**GDP** ** (US$ ×10^3^)**	**Latitude (°)**	**Muslims (%)**	**All medals**	**Predicted medals**	**Ratio all/** **predicted**
**Best medalists**
1	Ukraine	45	2.1	48	1.7	22	3.1	7.0
2	Kazakhstan	18	11	48	70	1	0.1	6.8
3	Slovakia	5.4	16	49	0.2	11	1.7	6.5
4	Belarus	9.5	5.9	54	0.8	12	2.6	4.7
5	Australia	24	57	−25	2.6	4	0.9	4.2
6	Austria	8.6	44	48	8.0	7	2.2	3.1
7	New Zealand	4.6	39	−41	0.9	3	1.0	2.9
8	France	67	37	46	8.8	20	7.7	2.6
9	Croatia	4.2	12	45	1.5	2	0.9	2.3
10	United States	321	57	37	1.1	36	18	2.0
**Worst medalists**
17	Neutral^a^	144	9.3	62	13	24	25	1.0
18	Italy	61	30	42	4.8	5	5.5	0.9
19	Korea	76	20	38	0.1	3	4.8	0.6
20	Spain	46	26	40	2.6	2	4.1	0.5
21	Finland	5.5	43	62	1.8	3	7.3	0.4
22	Great Britain	65	45	55	6.3	7	20	0.3
23	Belgium	11	41	51	7.6	1	3.5	0.3
24	Sweden	9.8	52	60	8.1	1	7.7	0.1
25	Poland	38	13	52	0.02	1	8.1	0.1
26	China	1,370	8.1	36	1.7	1	21	0.05
**Worst non-medalists**
82	Estonia	1.3	17	59	0.0	0	1.5	0
83	Latvia	2	14	57	0.2	0	1.6	0
84	India	1,310	1.6	21	15	0	1.6	0
85	Lithuania	2.9	14	55	0.1	0	1.8	0
86	Hungary	9.8	13	47	0.5	0	2.0	0
87	Romania	19.8	9.0	46	0.7	0	2.6	0
88	Argentina	43.1	14	−38	0.9	0	2.8	0
89	Czech Republic	10.5	18	50	0.2	0	3.1	0
90	Ireland	4.7	62	53	1.4	0	3.7	0
91	Denmark	5.7	53	56	5.4	0	4.3	0

a Russian athletes competed with this designation.

For medalists the ranking is highest to lowest values of the ratio all-events medals divided by medals predicted by population, gross domestic product per capita (GDP), absolute latitude and Muslim proportion; for non-medalists, the ranking is lowest to highest values of predicted medals.

**Table 10:** Ranking of the best medalists, worst medalists and worst non-medalists at the Tokyo summer Paralympics.

**Rank**	**Nation**	**Population (×10^6^)**	**GDP** ** (US$ ×10^3^)**	**Latitude (°)**	**Muslims (%)**	**All medals**	**Predicted medals**	**Ratio all/** **predicted**
**Best medalists**
1	Azerbaijan	9.8	4.1	40	97	19	2.7	7.0
2	Ukraine	45	2.6	48	1.7	98	15	6.4
3	Australia	25	54	−25	2.6	79	13	6.1
4	Colombia	49	6.4	5	0.2	24	4.4	5.4
5	Tunisia	11	3.5	34	100	11	2.2	5.0
6	Uzbekistan	32	1.8	41	89	19	5.3	3.6
7	Brazil	208	9.9	−14	0.4	72	23	3.2
8	Jordan	9.8	4.2	31	97	5	1.9	2.6
9	Algeria	41	4.1	28	98	12	5.0	2.4
10	Morocco	36	3.0	32	99	11	4.6	2.4
**Worst medalists**
77	Finland	5.5	46	62	1.8	5	16	0.3
78	Ethiopia	106	0.8	9	31	1	3.6	0.3
79	Peru	31	6.7	−9	0.0	1	3.8	0.3
80	Portugal	10	21	39	0.4	2	8.1	0.2
81	Norway	5.3	76	60	3.2	4	18	0.2
82	Romania	20	11	46	0.7	2	13	0.2
83	Saudi Arabia	33	21	24	98	1	6.8	0.1
84	Vietnam	95	2.4	14	0.1	1	7.1	0.1
85	Chinese Taipei	24	29	25	0.3	1	10	0.1
86	Pakistan	208	1.5	30	97	1	13	0.1
**Worst non-medalists**
196	Uruguay	3.4	19	−33	0.0	0	2.6	0
197	Iceland	0.3	72	65	0.2	0	2.6	0
198	Guatemala	17	4.5	16	0.0	0	2.6	0
199	Angola	30	4.1	−11	0.3	0	3.3	0
200	Nepal	28	1.0	28	4.2	0	3.5	0
201	Estonia	1.3	20	59	0.0	0	3.5	0
202	Syria	17	4.2	35	87	0	3.6	0
203	Myanmar	53	1.3	22	4.3	0	4.8	0
204	Philippines	105	3.1	13	8.0	0	7.9	0
205	Bangladesh	160	1.6	24	90	0	8.6	0

For medalists the ranking is highest to lowest values of the ratio all-events medals divided by medals predicted by population, gross domestic product per capita (GDP), absolute latitude and Muslim proportion; for non-medalists, the ranking is lowest to highest values of predicted medals.

### Olympic-Paralympic comparisons

After adjustment of medal tallies for the effects of population, wealth, latitude and Muslim proportion, nations that reached the top-10 medalists in the Olympic and Paralympic winter games were Austria, Belarus, Kazakhstan, Slovakia and the Ukraine, but only Azerbaijan reached the top 10 in both summer games. Nations in the bottom-10 medalists at both winter games were Belgium, China, Great Britain, Poland and Spain, while Finland, Romania and Saudi Arabia were in the bottom-10 at both summer games. Nations in the bottom-10 non-medalists at both winter games were Argentina, Denmark, Estonia, Ireland, Lithuania and Romania, while Bangladesh, Iceland and Uruguay were in the bottom-10 non-medalists at both summer games.

**Figure 3:**
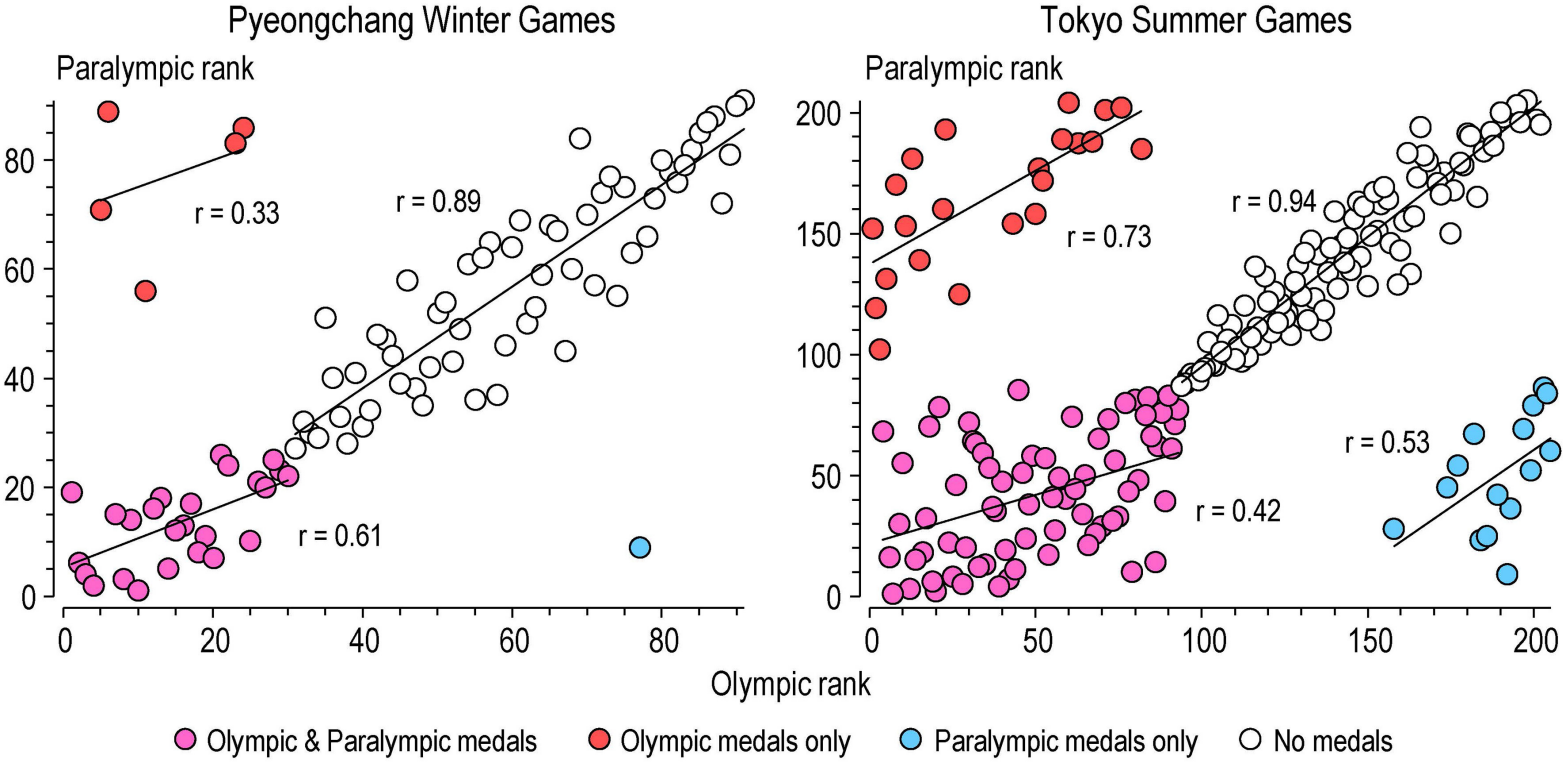
Relationships between Olympic and Paralympic medal ranks of nations that attended the Pyeongchang winter games and the Tokyo summer games. Regression lines and correlations are shown for nations grouped according to whether or not they won medals at the Olympics and Paralympics.

Figure 3 shows the relationships between Paralympic and Olympic nation ranks at the winter and summer games. The highest correlations (very large and extremely large) were for nations that won no medals. The other correlations show that worse medal and non-medal ranking at the Olympics was followed to some extent by worse rankings at the Paralympics. Amongst nations that failed to get any medals at the Olympics, only one (Croatia) medaled at the winter Paralympics, whereas 15 medaled at the summer Paralympics, one of which (Algeria) reached the top 10. Five nations at Pyeongchang and 22 at Tokyo won medals in the Olympics but failed to win medals in the Paralympics. Ukraine, Kazakhstan, Slovakia, Belarus, and Austria were in the top-10 list at both Olympic and Paralympic winter games, but only Azerbaijan made it into the top 10 at both summer games.

## Discussion

### Pyeongchang and Tokyo Olympics

Our analyses have revealed remarkably large positive effects of population, GDP, and latitude on medal counts in the four different kinds of event at the winter and summer Olympics. All these effects were well defined, and most were in excess of 10-fold increases when expressed as differences between nations differing by 2 SD of the predictor. We expected positive effects for each of these variables, on the basis of common sense and previously published findings, but it is still surprising that the effects are so strong, considering that they have been adjusted for each other. The effects of population were similar in summer and winter games, but the effects of GDP in winter were about twice those in summer, presumably reflecting the greater cost of participation in winter sports arising from equipment and travel to appropriate sports venues. These differences were also apparent in the coefficients expressed in percent per percent units: the values were generally less than 1.0, indicating that, for example, a doubling of population or wealth was associated with less than a doubling of medal counts, but medals counts for winter male, female and all events were in ~1:1 proportional relationships with wealth, and doubling of wealth was associated with much more than a doubling of medal counts in the winter mixed events.

Strong positive effects were expected for latitude on medal counts at the winter Olympics, for the obvious reason that access to snow and ice is easier in countries further from the Equator. Interestingly, the effects of latitude remained positive even for the summer Olympics, although reduced in magnitude by a factor of ~3. Countries further from the Equator evidently enjoy an advantage even in summer sports that is independent of the other predictors, presumably arising from greater popularity of such sports in more temperate climates. We anticipated that the effects of latitude on summer medals might be non-linear, with less positive or even negative effects at the highest latitudes, but the examination of residuals gave no indication of such non-linearity.

Muslim proportion had generally weaker effects than those of the other predictors, but they were all negative. The effects had inadequate precision in the winter games, and the effects of the other predictors also had less precision in winter, a consequence of the smaller numbers of nations and medals per nation at Pyeongchang compared with Tokyo. In the summer games, the Muslim effect also had inadequate precision for mixed events, a consequence of the relatively small number of these events. The Muslim effects were strongest for females and weakest for males, but even male Olympians from fully Muslim nations were likely to win fewer medals than those from fully non-Muslim. Given their uncertainties, the differences in the effects on male and female Olympians might not be repeated at future summer games, but they are certainly consistent with the constraints to participation in sport experienced by Muslim women (e.g., Marwat et al., 2014).

The ranking of nations after differences in population, GDP, latitude and proportion of Muslims are accounted for will doubtless give some people from smaller or less wealthy nations a satisfying sense of schadenfreude to see the nations that always top the medal tables appearing much further down. Nations that win only one or two medals can appear deservedly in the top-10; Kazakhstan and Grenada are striking examples in the winter and summer Olympics, respectively. The expected variation in a count of one or two medals is ~ ±1 medal (~ ±2 with the observed over-dispersion), so the ranking of some of these nations will change dramatically between Olympics. Rankings will be more stable with larger medals counts, so Austria, Norway and the Netherlands will likely again earn their places in the top 10 in future winter Olympics, as will Korea, depending on the host advantage and its decay. Kenya and Cuba are likely candidates for future top-10 lists in the summer games. Japan apparently did not benefit enough from the host advantage to reach this list, perhaps a consequence of the lack of fans (Pettigrew and Reiche, 2016) due to COVID-19. Nations such as Ireland and Denmark in the winter games would need as many as 10 medals to move from the bottom of the non-medalists to a reasonably respectable ranking amongst the medalists. Winter Olympic sports are presumably not popular in these countries; the same can be said for Vietnam and Chile in the summer games.

### Pyeongchang and Tokyo Paralympics

The effects of population, GDP, latitude, and Muslim proportion on medal winning at the winter and summer Paralympics were generally similar in magnitude to those at the Olympics and have the same interpretations: bigger populations produce more athletes, greater wealth provides more funding for the athletes to train and compete, higher latitudes are more conducive to participation in summer and especially winter Paralympic sports, and Islamic culture is less supportive of sport. The effects of GDP were considerably smaller at the Paralympics than at the Olympics in all event groups, especially in the winter games. There is no obvious explanation for this difference, but it means wealth is less of a barrier to winning Paralympic medals.

In spite of the huge negative effect of Muslim proportion on total medals at the winter Paralympics, only one predominantly Muslim nation (Kazakhstan) was amongst the top-10 medaling nations, for the simple reason that it was the only predominantly Muslim nation to win any medals at the winter Paralympics. The effect of Muslim proportion resulted in no predominantly Muslim nation appearing in the bottom 10 of non-medalists. In the summer Paralympics, many Muslim nations won medals, and six of them appeared in the top-10 list, even though the effect was only moderately negative in the summer games. As we noted for the Olympics, it would be desirable to see these and other Muslim nations achieving relatively high medal counts in future Paralympics independent of their Islamic heritage.

The strong relationship between rankings for nations that won no medals in Olympics and Paralympics shows that the prediction equations give relatively similar weights to each predictor in the Olympics and Paralympics. The considerable sampling variation that occurs with low counts (especially with over-dispersion) is responsible for the greater scatter in the relationships with nations that won medals in either or both Olympics and Paralympics than with nations that won no medals. The scatter in these plots shows that medal performance at the Pyeongchang and Tokyo Olympics was not followed closely by Paralympic medal performance.

### Future research

We undertook this study in the first instance to estimate and adjust for the two effects that are most interesting to the wider public, whenever nations' medal hauls are presented in the popular media: population and wealth. We added two more that would be of interest to the public as conferring advantages or disadvantages for medal winning: geographical latitude (a proxy for weather, interesting from the perspective of summer vs. winter sports), and the proportion of Muslims (a proxy for Muslim culture, interesting for its possible effects on women's and mixed sports). Other sociodemographic factors could be added in future research, particularly indices of wealth inequality and gender inequality, which had substantial effects at the 2012 Winter and 2014 Summer Olympics (Berdahl et al., 2015). The effects of all the sociodemographic factors on medal winning in the different types of Olympic sport would also be worth investigating.

## Conclusion

Adjusting Olympic medal counts for demographic and geographic factors that are beyond the control of individual nations provides another dimension of entertainment for the Olympics and Paralympics. More importantly, the notion of a “level playing field” is fundamental to the Olympic ideal of fair play when athletes compete against each other, so when nations compete against each other for medal counts, adjusting the counts for factors beyond their control provides a comparison of nations' sporting prowess or engagement that is more in keeping with the Olympic ideal. Such comparison should also be more useful for nations that base their Olympic-funding decisions on medal performance, including those nations that are too small or poor to have much chance of winning any medals.

Perhaps the most important finding in the present study is the substantial and generally large effects of Muslim proportion on medal winning, especially in the women's and mixed events in the Tokyo Olympics. These effects are independent of the population, wealth and latitude of those nations, so they represent reasonably good evidence of the consequences of Islamic culture on Olympic sport performance. Adjustment for the effects of Muslim proportion contributed to elevating two predominantly Muslim nations to the top-10 list at the Tokyo Olympics and six at the Tokyo Paralympics, which represent their sporting prowess and engagement. But in the long term, it would be desirable to see these and other countries with predominantly Muslim population achieving relatively high medal counts in summer and winter games.

## Data availability statement

The original contributions presented in the study are included in the article/Supplementary material, further inquiries can be directed to the corresponding authors.

## Author contributions

WH: conceptualization and analysis. PL, FL, and WH: literature search, draft preparation, and editing. All authors contributed to the article and approved the final version.

## Conflict of interest

The authors declare that the research was conducted in the absence of any commercial or financial relationships that could be construed as a potential conflict of interest.

## Publisher's note

All claims expressed in this article are solely those of the authors and do not necessarily represent those of their affiliated organizations, or those of the publisher, the editors and the reviewers. Any product that may be evaluated in this article, or claim that may be made by its manufacturer, is not guaranteed or endorsed by the publisher.
